# YouTube Videos as a Source of Information on Colorectal Cancer: What Do Our Patients Learn?

**DOI:** 10.1007/s13187-018-1422-9

**Published:** 2018-09-21

**Authors:** Alain Nathan Sahin, Anne Sarah Sahin, Frank Schwenter, Herawaty Sebajang

**Affiliations:** 1 Department of Surgery, Centre Hospitalier de l’Université de Montréal, 1051 rue Sanguinet, Montréal, Québec H2X 0C1 Canada; 2 Department of Surgery, Université de Montréal, C.P. 6128, succursale Centre-ville, Montréal, Québec H3C 3J7 Canada

**Keywords:** eHealth, YouTube, Informatics, Social media, Colon cancer

## Abstract

YouTube is the second most visited website in the world. No studies to date have characterized and evaluated YouTube videos on colorectal cancer (CRC) although these videos could influence patient decision-making, notably regarding screening and prevention. This study aims to report the characteristics and quality of these videos as patient education resources for CRC. YouTube’s search engine was queried with different search phrases relating to CRC. The first two pages of each search result were analyzed. Two specialists devised a critical appraisal tool with a list of criteria to assess the videos. Quantitative YouTube parameter analyses and criteria assessment were performed. Inter-rater agreement was assessed between three raters. A total of 46 videos were eligible to be included in the study. The videos were on average 4:51 ± 3:27 min long. The videos had 10 times as many likes as dislikes. Less than half the videos discussed risk factors and protective factors. Only 41% of videos mentioned screening tests and only about a quarter discussed them. Palliative care was only mentioned in 2% of videos. A single video could obtain a perfect score on the critical appraisal tool. Length was the unique parameter associated with a high score on the criteria list. There is thus a need for more authoritative and comprehensive videos easily identifiable by the patients. Video popularity is not associated with comprehensiveness. Currently, YouTube might not be an education resource for CRC suited to every patient.

## Introduction

Eight in ten Internet users consult the Web to search for health information [1, 2]. In fact, personal research online can be empowering and educational, and is frequently encouraged [3–6]. A study from 2000 found that 70% of users who sought health information online reported that the Web affected their decision-making process [7, 8]. In 2005, the Health Information National Trends Survey revealed nearly half of individuals looking for cancer information used the Internet as a first resort instead of asking their physicians [6, 9]. In parallel, it is known that health professionals must understand their patients’ views and fears to better address them and guide them towards proper educational resources [3, 6]. This patient-centered care approach leads to better patient satisfaction and health outcomes [10].

eHealth studies can help assess the quality of patient educational content online and understand the views of the patients who use these resources [11, 12]. Currently, YouTube is the second most visited website in the world after Google and contains 60% of all videos stored on the Internet [13–15]. Several studies have evaluated the content of YouTube videos with respect to their specific topics of interest and have been cited widely [15–19]. A 2012 systematic review of these studies showed that YouTube videos contain misleading or biased information and highlighted the importance of designing interventions to guide eHealth consumers [16]. It is important to assess whether the information delivered by videos on colorectal cancer is accurate or misleading as patients, relatives, and other members of their community may consult them in the hopes of better understanding the disease and/or taking informed decisions.

In the United States, colorectal cancer is the second most common cause of cancer mortality [20, 21]. Screening effectively decreases the mortality of colorectal cancer by early intervention [22]; however, in 2013, more than 40% of patients who fit the screening guideline recommendations did not undergo testing [22]. Thus, the National Colorectal Cancer Roundtable has set the goal of achieving 80% screening rates in the United States [22]. Barriers to screening include misconceptions about risk factors and screening techniques [23, 24]. More specifically, commonly cited barriers to colonoscopy or sigmoidoscopy are fear of these procedures and the required bowel preparation [25–27]. Certain patients fear that physicians seek financial profit by suggesting these procedures [26, 27]. Screening programs must therefore be tailored to the patients’ beliefs [26].

In the past, a study has looked at online resources for colorectal cancer [2]: Wasserman et al. found that sources were frequently incomplete and that their quality was variable [2]. Even though 19% of the websites that the study evaluated were created by professional or medical societies, the authors believed that patients could not make informed decisions based on online resources and encouraged organizations to improve online content [2]. Nevertheless, Wasserman et al. excluded all YouTube videos from their analysis and their study dates from 2014. The purpose of this study was to describe the characteristics of YouTube videos and to assess their quality as patient education resources for colorectal cancer.

## Methods

Our methodology is based on previous studies [14, 15, 17, 21, 28] and on published design suggestions [11, 16]. YouTube’s search engine was queried with three distinct key phrases in order to identify relevant videos: “colorectal cancer,” “colon cancer,” and “bowel cancer.” The search was conducted on March 3, 2018. Because 62% of users pick entries in the first page of search result in similar settings [2], we have reviewed the first two pages of search results (40 pages per phrase) similar to what other studies have done [14, 29]. Exclusion criteria included videos not in English, news stories, patient testimonials, duration > 15 min, and duplicates. The study on YouTube prostate cancer videos only studied videos shorter than 10 min [15]. Information science studies have shown users of search engines usually do not consult more than five results and want to access the information they want within 15 min, after which they put a stop to their activity [30]. An analysis of videos on edX, the massive open online course provider created by the Massachusetts Institute of Technology and Harvard University, has similarly noted that the ratio (time spent watching the video/length of the video) is low after the 15-min mark [31]. We believe that patients would watch longer videos if these videos were specifically recommended by healthcare professionals. However, YouTube is currently not highly integrated in health promotion interventions, as noted by other studies [32, 33], because of the lack of research in this domain. This study meets the exclusion criteria of the Canadian Tri-Council Policy Statement [34] for research as an observational study with no human participants. Institutional research ethics review board is not required for this type of research.

Many methodological elements frequently collected in YouTube studies can be classified as popularity-driven measures or heuristic-driven measures [11, 16]. The number of views, comments, likes, dislikes, and the number of subscribers to the channel would be classified as popularity-driven measures [11]. Likewise, the length of the videos and the date of upload would be classified as heuristic-driven measures [11]. In this study, for each video, the length, the view counts, the number of comments, the number of likes, the number of dislikes, the country of origin, the date of the upload, and the number of subscribers to the channel were noted. If information was missing due to the channel creator restricting them, they were not taken into account in their corresponding analysis. Regarding the upload source, we identified channels that would be considered authoritative. These consisted of professional or medical organizations. For example, we coded the channels “Stanford Health Care” and “Mayo Clinic” as authoritative. However, we did not consider channels from small-scale private clinics or for-profit companies to be authoritative.

Currently, no validated tool suitable for the objectives of our study exists for the evaluation of these videos [28]. Two attending physicians practicing in a quaternary center and a student in his third year of medical studies devised a list of assessment criteria for the videos. Similar to other studies [14, 21], a point-based rating tool was constructed. This tool had a total of 12 evaluation criteria that were each given the rating of 0 or 1 depending on the satisfaction of the assessment criterion. Any misleading or false information from a video resulted in a rating of 0 for the corresponding criterion because of the continued influence effect [35] and high diffusion of science news misinformation [36]. The criteria were the following: (1) portrays basic microscopic or macroscopic anatomy; (2) lists risk factors or preventive factors; (3) lists signs or symptoms; (4) mentions available screening tests; (5) discusses available screening tests; (6) mentions available diagnostic tests; (7) discusses available diagnostic tests; (8) explains staging of colon cancer; (9) discusses prognosis; (10) mentions treatment options; (11) discusses treatment options; and (12) mentions palliative care. Fulfilling criteria 5, 7, and 11 would by extension give an additional three points for criteria 4, 6, and 10 as a discussion entails a more detailed treatment of those topics. We define a low-score video as a video that has a summative score of less than six (< 6). Higher scores entail a video that encompasses several content foci and that is more comprehensive. We designed this tool to comprehend all three levels of prevention (primary, secondary, and tertiary). To our knowledge, this is the first tool to identify high-quality comprehensive videos on colorectal cancer.

The first author screened all videos and analyzed the ones of interest. The two physicians also independently evaluated a sample of videos. To assess total score reliability, the intraclass correlation coefficient was calculated to validate inter-rater agreement using a single-measurement, absolute-agreement, two-way random-effects model. The intraclass correlation coefficient among the three raters was 0.72, showing good agreement. Bivariate analysis using Mann-Whitney *U* tests was performed to determine differences in videos classified as useful (defined as satisfying at least six criteria in this study), misleading, or from authoritative sources. A summary criteria compliance table was created to see the proportion of the videos that satisfy each criterion.

## Results

**Fig. 1:**
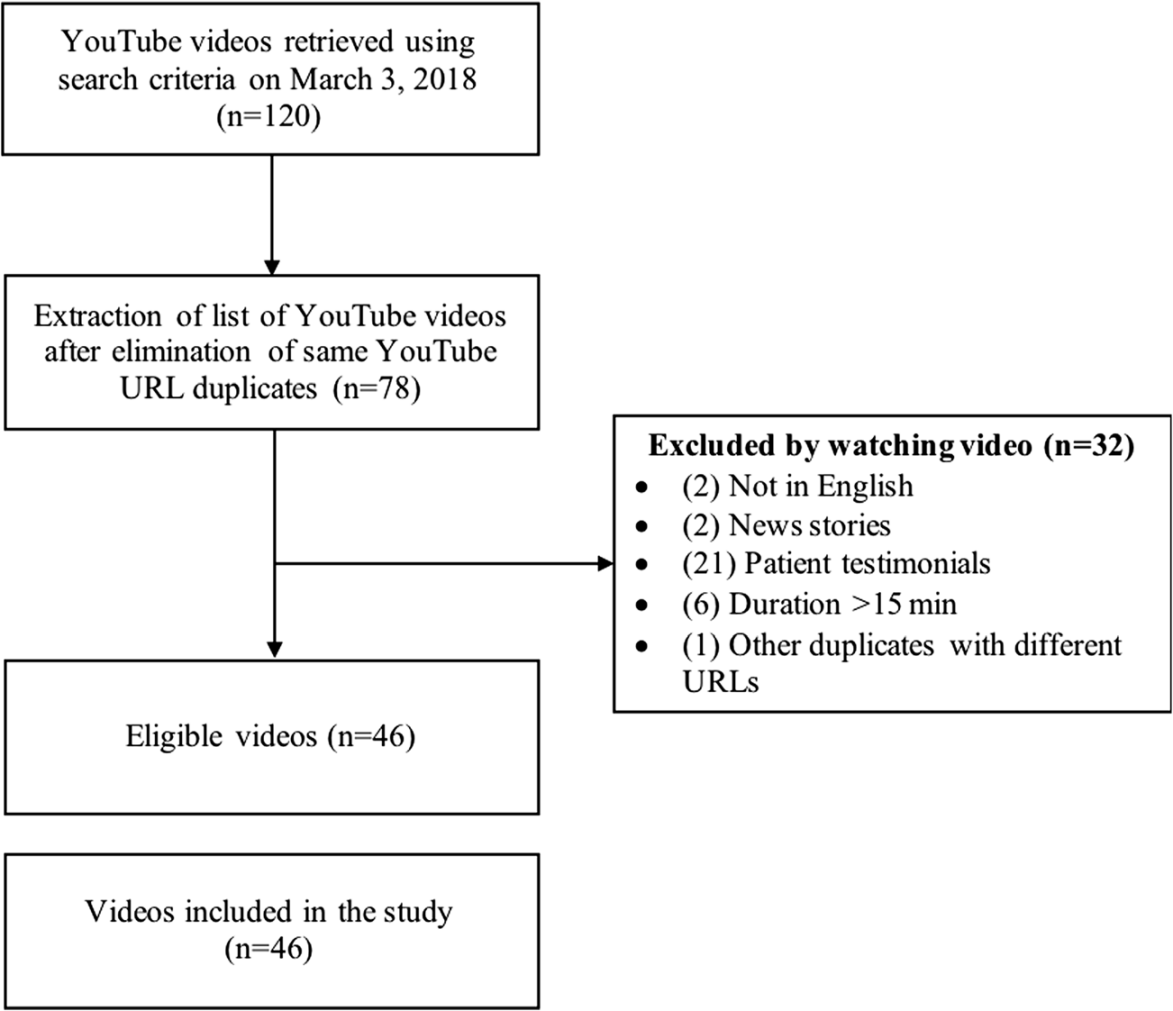
Flow chart of the search results and screening process

Our study included 46 videos. Of the identified videos using the specified search phrases, 27% were patient testimonials. Figure 1 presents the screening process.

Table 1 summarizes the descriptive statistics measures on the videos and shows the results of bivariate analysis on the quantitative parameters. Using rounded values, the average video lasted 6 min, had 200,000 viewers, was commented on 65 times, was liked by 650 users, was disliked by 60 accounts, came from a channel with a considerable number of subscribers (350,000), and was uploaded recently in the past 2.5 years. Popularity-driven measures have larger coefficients of variation than heuristic-driven measures. YouTube’s search engine hence allows users to access less popular videos within the first pages of results. Only length was significantly associated with criteria satisfaction (*p* = 0.03). Shorter videos thus have lower scores.

**Table 1:** Quantitative characteristics of YouTube videos on colorectal cancer and bivariate analysis

	Mean for all videos (*n* = 46)	Range for all videos	Low-score videos (*n* = 35)	High-score videos (*n* = 11)	*P* value
Length (seconds)	351 ± 207	20–724	255 ± 197	408 ± 206	0.03
View counts	194,923 ± 540,611	27–3,039,975	240,583 ± 618,782	113,440 ± 148,677	0.74
Number of comments	55 ± 126	0–496	52 ± 120	66 ± 153	0.51
Number of likes	647 ± 1799	0–8878	542 ± 1575	981 ± 2442	0.29
Number of dislikes	62 ± 182	0–970	74 ± 207	26 ± 40	0.63
Number of subscribers to the channel	337,489 ± 1,016,581	19–6,042,273	201,950 ± 538,270	698,960 ± 1,788,345	0.31
Days since upload	948 ± 897	0–4227	965 ± 927	895 ± 834	0.74

Low score defined as < 6 satisfied criteria. Data reported as mean ± standard deviation

With regard to the countries of origin of the eligible videos, the USA was the country that produced the largest number of videos on colorectal cancer (46%). Other videos mainly came from the UK (15%), India (9%), Australia (6%), and Nigeria (6%). The rest of the videos either came from other countries (11%) or the country of origin was not listed (7%).

Table 2 presents the proportion of videos satisfying each criterion specifically. Less than half of videos listed risk factors or preventive factors (48%). Only 41% of videos mentioned available screening tests. Twenty-six percent of videos discussed these screening tests. A mere 2% of videos mentioned palliative care. By analyzing the number of criteria satisfied by each video, we found that less than a quarter of videos (24%) satisfied at least half of the established criteria, with the mode being three. A single video could satisfy all 12 criteria.

**Table 2:** Evaluation criteria and compliance proportions

Evaluation criteria	Proportion of videos that satisfy the criteria (%)
Portrays basic microscopic or macroscopic anatomy	54
Lists risk factors or preventive factors	48
Lists signs or symptoms	48
Mentions available screening tests	41
Discusses available screening tests	26
Mentions available diagnostic tests	30
Discusses available diagnostic tests	20
Explains staging of colon cancer	13
Discusses prognosis	24
Mentions treatment options	33
Discusses treatment options	26
Mentions palliative care	2

Out of the 46 retained videos, seven offered misleading or non-evidence-based information to patients. These could notably overestimate the potential benefits of alternative medicine or confuse pathologies. Examples include statements such as “Studies have found that drinking green tea regularly reduces the risk of colorectal cancer by 50%” or “EVEN THE DOCTORS ARE SHOCKED [coconut oil] Kills 93% of Colon Cancer.” In another case, one video was entitled “Colon Cancer”, but only discussed cap polyposis, a different colorectal disease.

Of note, regarding their number of views, these misleading videos had, on average, five times the average number of views of all videos (misleading and others combined). The misleading videos had the following number of views, likes, and dislikes, respectively: 962,824 ± 1,222,232; 2226 ± 3112; 338 ± 374. The values for the other videos were as follows: 66,940 ± 104,979; 363 ± 1326; 12 ± 24. The average total score of the misleading videos was 0.7 ± 1.0, whereas the average for the other videos was 4.2 ± 3.0. All *p* values for these comparisons between the misleading videos and the other videos were statistically significant (*p* < 0.01). Consequently, misleading videos have more views, are rated more frequently by viewers, and have lower total scores.

The authoritative channels uploaded a total of 14 videos. Although the videos from these channels had a higher average total score (4.6 ± 3.1) compared to the other videos (3.3 ± 3.0), this difference was not statistically significant (*p* = 0.16). In spite of that, none of the 14 videos were deemed to be misleading.

## Discussion

YouTube is increasingly becoming accessible and viewed for health information retrieval by patients [14, 37]. Care providers from across disciplines and fields of medicine should understand how patients might consult misleading or limited sources of information and notably not know of or understand colorectal cancer screening benefits. This study has identified, analyzed, and evaluated the most relevant videos on YouTube on colorectal cancer. Our main findings were that there is a sparse number of comprehensive videos on colorectal cancer on YouTube and that none of the popularity parameters are associated with comprehensiveness of the videos.

The results of this research are relevant for preventive healthcare. For example, half of the videos listed risk factors or preventive measures. Only about the quarter discussed screening methods. Less than a third mentioned the diagnostic tests. A third mentioned treatment options and about a quarter discussed them. Finally, only a single video mentioned palliative care. We believe that mentioning that there are screening tests available and discussing the indications of screening would be relevant and beneficial in any video on colon cancer.

Patients with different health literacy levels might struggle to acquire knowledge from these videos. Ratzan and Parker defined health literacy to be “The degree to which individuals have the capacity to obtain, process, and understand basic health information and services needed to make appropriate health decisions” [38, 39]. As such, the paucity of high-quality information and the variability of source credibility raise concern, because these two factors affect information retrieval, information analysis, and decision-making processes of patients. In addition, authoritative organizations and awareness campaigns should consider uploading their videos on YouTube. Of note, government-sponsored videos from other Anglophone countries such as Canada, Ireland, and New Zealand could not be found using our screening process. Because length was significantly associated with comprehensiveness, a balance needs to be reached between comprehensiveness and the attention span of the viewers. It is also possible that videos longer than 15 min are more comprehensive. Care providers should also be aware that we could not find an association between popularity measures and information content, a finding consistent with a similar study on prostate cancer [15]. The videos had on average 10 times as many likes as dislikes. Although this might represent appreciation by the viewers, it may be that viewers who do not like the video simply directly go looking for other videos. Presently, we suggest that care providers discuss what patients learned from these resources to make sure those with low health literacy levels make informed health decisions.

Simple steps can be taken to enhance the accessibility of videos for patients. As the YouTube search engine is by default set to rank the uploaded videos based on “relevance,” generating video metadata is essential; this includes putting enough relevant tags on the video, using keywords in the title and description, and choosing a representative thumbnail. To enhance accessibility for users whose mother tongue is not English or for deaf sign language users, video uploaders should consider adding subtitles through YouTube’s interface. Moreover, some videos only consisted of text being displayed and music being played in the background. Finally, color contrasts, video resolution, and sound quality must be adequate.

Previous research has shown that patient testimonials affect patient decision-making [16, 40–43], but certain patient testimonials on YouTube may be anecdotal or biased and lack important information for informed consent [16, 42]. Following emerging models of patient partnerships [44, 45] where the patient’s life story enables him or her to teach others about the disease, future research should also investigate how informative patient testimonials are in comparison to authoritative references. The search patterns of patients on online tools with specific emphasis on the keywords they use should be ascertained. Likewise, we should investigate the patients’ appreciation of videos that score highly on the expert-created critical appraisal tool. Lastly, future work should gauge the extent of the impact of colorectal cancer YouTube videos on patient decisions.

There are limitations to our study. First, our rating score tool is not validated albeit being reproducible. Furthermore, YouTube is a dynamic participatory forum. Our study is cross-sectional in nature and might not reflect the evolving nature of the site. We also could have proceeded to qualitative theme-based analysis of the videos. Moreover, our study was limited to English videos. Finally, a formal analysis of the accessibility of YouTube videos, similar to other evaluations of patient education resources [7, 46, 47], would be of benefit.

In this study, we have analyzed the videos on colorectal cancer available on YouTube. In the era of Web 2.0 [48], i.e., participatory Web, users frequently access, use, and disseminate online health information. If integrated appropriately in a clinical setting, YouTube could act as an effective user-friendly learning interface for colorectal cancer patients and their families. A previous YouTube study on cataract surgery has recommended that health care professionals direct patients to specific highly rated videos [14]. However, health care providers and organizations must be aware of the limitations and pitfalls of these platforms to address them appropriately in patient education and care.
